# Progestin-primed ovarian stimulation protocol in patients undergoing assisted reproductive technology

**DOI:** 10.3389/frph.2025.1719930

**Published:** 2026-01-23

**Authors:** Shanqin Qi, Haiyan Yu, Xiaojing Yang, Qinghan Shi, Liu Yang, Kehua Wang

**Affiliations:** 1 The First Clinical Medical College, Shandong University of Traditional Chinese Medicine, Jinan, Shandong, China; 2 Department of Reproductive Medicine, The Affiliated Taian City Central Hospital of Qingdao University, Taian, Shandong, China; 3 Department of Obstetrics, The Affiliated Taian City Central Hospital of Qingdao University, Taian, Shandong, China; 4 Department of Gynecology, The Affiliated Taian City Central Hospital of Qingdao University, Taian, Shandong, China; 5 Department of Preventive Health Care of Traditional Chinese Medicine, Tai’an Hospital of Traditional Chinese Medicine, Taian, Shandong, China; 6 Reproduction and Genetics Center, Affiliated Hospital of Shandong University of Traditional Chinese Medicine, Jinan, Shandong, China

**Keywords:** embryo competence, *in vitro* fertilization/intracytoplasmic sperm injection (IVF/ICSI), oocyte quality, premature LH surge, reproductive outcome

## Abstract

**Purpose:**

To evaluate the effectiveness and safety of the novel protocol—progestin-primed ovarian stimulation (PPOS) protocol during controlled ovarian hyperstimulation (COH), in patients undergoing *in vitro* fertilization/intracytopalsmic sperm injection and embryo transfer (IVF/ICSI-ET).

**Methods:**

By reviewing and analyzing published studies since PPOS protocol was firstly reported in 2015, we compared differences in ovarian stimulation characteristics, embryological features, pregnancy rates, and neonatal outcomes between PPOS protocol and conventional regimens employed in assisted reproductive technology (ART), and discussed the advantages and limitations of PPOS protocol.

**Main finding:**

By adding exogenous progestin (P) during early follicular phase, PPOS scheme provide robust control over preovulatory luteinizing hormone (LH) surge and spontaneous ovulation, which promote oocyte maturation and recovery. Compared to various traditional protocols, PPOS achieved promising clinical pregnancy results, and equivalent rates of birth defect and congenital malformation. Moreover, it possessed significantly lower risk of ovarian hyperstimlation syndrome (OHSS).

**Conclusion:**

Not inferior or comparable outcomes indicated that PPOS protocol is a competent alternative for ART with no obviously detrimental impact on oocyte development and embryo quality.

## 1 Introduction

One of the premises of a successful *in vitro* fertilization/intracytoplasmic sperm injection and embryo transfer (IVF/ICSI-ET) treatment is high-quality oocytes and embryos. The suggested way to improve pregnancy outcomes is to facilitate the growth and maturation of follicles and ensure the generation of viable or high-quality embryos (1). The rise in the serum estradiol (E2) concentration with multi-follicular development results in the premature surge of the luteinizing hormone (LH) via positive feedback effects on the pituitary level to induce ovulation. This reduces the number of oocytes collected and disrupts the schedule of assisted reproductive technology (ART), leading to disappointing results for both clinicians and patients (2, 3). Gonadotropin-releasing hormone (GnRH) analog is extensively used to suppress untimely LH surges, however, due to the complexity of treatments, specific side effects, and ineffective blockade of GnRH analog (4–8), better approaches are urgently needed to control preovulatory LH surges.

Kuang and colleagues (9) successfully conducted luteal phase stimulation and showed that luteal phase endogenous progesterone blocks the LH surge. The same group (10) investigated the novel progestin-primed ovarian stimulation (PPOS) protocol to explore the effect of follicular phase exogenous progesterone in inhibiting untimely LH rise. The PPOS protocol showed similar outcomes to the traditional controlled ovarian stimulation (COS) regimens (10–16), and the several advantages of PPOS over the classical method, including oral administration, lower cost, and good compliance, make it a widely used approach (12, 17).

PPOS protocol simultaneously combines conventional COH medications including but not limited to gonadotropin (Gn), commonly known as human menopausal gonadotropin (hMG) and follicle-stimulating hormone (FSH) (17–19), or GnRH analog (20–22), or clomiphene citrate and letrozole (23–26), with progestin, such as medroxyprogesterone acetate (MPA) (10, 17, 21, 27–30), dydrogesterone (Duphaston, DYG) (18, 20, 29–31), or micronized progesterone (Utrogestan, MIP) (32, 33), from the natural or artificial menstruation cycle day (MC) 2–5 to human chorionic gonadotropin (hCG) administration day. The final stage of oocyte maturation is induced by GnRH agonist (GnRHa) alone or a co-trigger with low-dose hCG. After oocyte pick-up and fertilization by IVF or ICSI, cryopreservation of the whole embryo cohort and a delayed freeze-thaw embryo transfer (FET) are carried out sequentially (10, 18, 29).

In a natural cycle, as multiple follicles develop, estradiol is continuously produced and induces an LH surge in the late follicular phase leading to spontaneous ovulation (34). Progestin was originally used for contraception as it resists the aforementioned procedure. Later, it was found to be effective in preventing premature ovulation in COH (31, 35). When progestin is continuously given (starting from the early follicular phase), it can inhibit the LH peak and allow more oocytes maturation and retrieval by blocking the estradiol-induced positive feedback of GnRH pulse and Gn secretion through hypothalamus-pituitary-ovary (HPO) axis (36). However, early exposure of the endometrium to progestin leads to reduced receptivity and embryo-endometrium asynchrony; fresh embryo transfer is not feasible in this context and FET in a later cycle is required (35, 37, 38). Advancements in cryopreservation successfully separate the standard sequence of stimulation-retrieval-fertilization-transfer (12), which promoted the widespread acceptance of the PPOS scheme (17).

This article extensively reviewed studies and compared the efficacy and safety of PPOS protocol with conventional protocols. The comparative parameters include ovarian stimulation characteristics, embryonic features, pregnancy rates, and neonatal health risks in ART.

## 2 Materials and methods

### 2.1 Literature search

We mainly searched two databases, PubMed and the Cochrane Library, covering the period from 2015 to 2024. A combined search was carried out using MeSH terms and free-text keywords, including but not limited to “progestin-primed ovarian stimulation”, “PPOS”, “controlled ovarian hyperstimulation”, “COH”, “controlled ovarian stimulation”, “COS”, “*in vitro* fertilization”, “IVF”, “assisted reproductive technology”, “ART”. Relevant references were also manually searched. After deleting duplicates and reading through the entire text, we selected 29 articles on the clinical research of the PPOS protocol and extracted information regarding the mechanisms and results of PPOS.

### 2.2 Selection criteria

Inclusion criteria: (1) RCT or retrospective studies published in English; (2) undergoing IVF or ICSI; (3) study group is the PPOS regimen, control group is GnRH analog regimen, natural cycle, or micro-stimulation regimen; (4) research indicators involve COS data, IVF laboratory results, and pregnancy outcomes.

Exclusion criteria: (1) narrative reviews, conferences, etc.; (2) missing original data.

### 2.3 Risk assessment

Two authors (SQ and HY) assessed the risk of bias for each literature using the Cochrane bias risk tool, categorized into three levels: low risk, medium risk, and high risk. If there was a disagreement, it was discussed and decided with the third author (XY).

## 3 Results

### 3.1 Ovarian stimulation, oocyte performance, embryonic feature, and clinical outcome

#### 3.1.1 Comparison with GnRHa short protocol

Kuang et al. (10), for the first time, conducted a prospective controlled study consisting of 300 normogonadotropic patients to compare the IVF/ICSI outcomes with PPOS and GnRHa short protocol. The number of retrieved oocytes through the PPOS protocol was similar to the GnRHa short protocol (9.9 ± 6.7 vs. 9.0 ± 6.0), however, PPOS resulted in higher hMG consumption and persisted LH suppression. Premature LH surge was 0.7% in the PPOS group. The implantation rate (IR, 31.9% vs. 27.7%), clinical pregnancy rate (CPR, 47.8% vs. 43.3%), and live birth rate (LBR, 42.6% vs. 35.5%) in the PPOS group were slightly higher than that of the controls but failed to reach a statistical significance. They concluded that MPA was a viable alternative for LH surge suppression in patients undergoing COH. Zhu et al. (33) replaced MPA with MIP and got similar results, i.e., comparable oocyte retrieval, mature oocyte rate, IR, and CPR. Wang et al. (17) verified the protocol in polycystic ovarian syndrome (PCOS) patients and achieved a higher fertilization rate (77.69 ± 16.59 vs. 70.54 ± 19.23) and ongoing pregnancy rate (OPR, 58.67% vs. 42.86%) in the PPOS group. Wen et al. (39) detected 12 elevated lipidomic components in follicular fluid (FF) in patients receiving PPOS, and this group showed a better clinical outcome compared with the patients on the short GnRHa protocol.

#### 3.1.2 Comparison with GnRH antagonist protocol

The GnRH antagonist (GnRHant) protocol has gained steady popularity since the 1990s (40, 41). This protocol is flexible, swift, and includes reversible suppression of the pituitary, so the comparisons of the PPOS protocol with the GnRHant protocol are quite common. In a retrospective analysis, Huang et al. (42) concluded that among patients with poor ovarian response (POR), the PPOS group had significantly higher rates of metaphase II (MII) oocytes, fertilization, top-quality embryo, clinical pregnancy, and live birth compared with the antagonist group. Du et al. (43) applied PPOS in patients with POR diagnosed according to Poseidon standard, and found that the Gn dosage of PPOS protocol in Poseidon 1 group was higher than that of antagonist protocol, but the number of available embryos in Poseidon 2 groups was significantly increased, and other outcomes were similar. Yildiz et al. (11) performed a self-control study involving two COH cycles within 6 months per oocyte donor (a flexible PPOS cycle following a previous antagonist cycle) in which MPA was administered based on the same standard as the antagonist. More cumulus-oocyte complexes (COC) and more MII oocytes were collected from the flexible PPOS group than from the control. Recipients achieved identical rates of fertilization and pregnancy. This study was considered to be a high-quality analysis and support the effectiveness of the PPOS protocol in a systematic review (12). After that, the same team used flexible PPOS protocol in decreased ovarian reserve (DOR) patients, and achieved identical results as flexible antagonist scheme in terms of premature LH rate, number of retrieved eggs and MII eggs; They also concluded that fPPOS has less inhibition on pituitary and better response to GnRHa trigger in normal responders (22, 44, 45). Li et al. (46) implemented a prospective cohort study to explore the differences in apoptotic rates of granulosa cells (GCs) and hormone levels in FF between patients on the PPOS and the antagonist protocol. They obtained similar GCs apoptotic rates, FF hormonal profiles, ovarian response, and laboratory parameters, including MII oocyte rate, fertilization rate, and optimal-embryo rate. Several other studies (10, 13, 16, 19, 20) from different countries and centers also independently arrived at similar conclusions that the PPOS protocol is as effective as the antagonist protocol in providing COH.

In contrast, Beguería et al. (21) achieved an unexpectedly lower biochemical pregnancy rate (BPR), CPR, OPR, and LBR in the MPA group compared with the antagonist group in a prospective randomized controlled trial (RCT). They speculated that the primary reason for this discrepancy is that the recombinant FSH (rFSH) administered in their study was hCG free, while the hMG used in some studies (47) contained microdose hCG, which improved LH activity. They also triggered ovulation with GnRHa alone, which may be less effective than co-trigger with GnRHa and low-dose hCG (21). Notably, ovarian stimulation, laboratory features, and recipients' baselines were all comparable between the two groups. The inferior pregnancy rates may be because of uncontrolled confounders or the protocol itself and need to be further analyzed. Caetano et al. (48) recovered less oocytes (mean number 9 vs. 11) and slightly higher CPR (58.4% vs. 54.9%, *p* = 0.735) in the progestin (MPA or DYG) group compared with the GnRHant group. The authors declared that the oocyte collection difference might be attributed to the baseline heterogeneity. The GnRHant group showed lower baseline FSH and more antral follicle count (AFC), indicating better ovarian reserve. No significant differences were observed in gonadotropin dose and duration, fertilization rate, and blastocyst formation rate, and the LH blockade was credible (< 1%).

#### 3.1.3 Comparison with GnRHa long protocol

Chen et al. (13) performed a self-controlled, retrospective study to investigate the pregnancy rate of PPOS protocol in aged infertile women who had a failed previous cycle with GnRHa long protocol. They found significantly higher oocyte utilization rate (54.5% vs. 46.9%) and optimal embryo rate (59.9% vs. 45.6%) and correspondingly increased CPR (22.58% vs. 12.70%) after FET in the MPA group.

#### 3.1.4 Comparison with GnRHa ultra-long protocol

Yang et al. (14) compared the effect of PPOS with GnRHant and GnRHa ultra-long protocol in endometrioma patients undergoing IVF/ICSI. In this study, BPR, CPR, and LBR were significantly lower in the MPA group than that in the ultra-long group (OR 2.3, 95% CI 1.1–4.9, OR 2.4, 95% CI 1.1–5.3, OR 2.6, 95% CI 1.3–5.1, respectively) but were all similar to the GnRHant group. Oocyte collection, endometrial thickness, and OPR were equivalent in all arms. Noticeably, demographic data were balanced between PPOS and GnRHant groups. But the ultra-long group had a significantly lower age of patients (30 vs. 34) and partners (31 vs. 34), and more AFC (11 vs. 5), these may be the contributing factors to the discrepancy in pregnancy rates.

#### 3.1.5 Comparison with mild stimulation protocol

Mild stimulation has been proved to generate high-quality oocytes and fewer aneuploid embryos due to less intervention to the ovary. This was especially important for aged or diminished ovarian reserve (DOR) patients, therefore, someone (49) chose it as control while validating the PPOS protocol. Peng et al. (50) investigated the embryological characteristics between the two regimens in women aged above 40. They found a significantly lower spontaneous LH surge rate (2.1% vs. 12.3%) and a higher percentage of top-quality embryos (50.08% vs. 33.29%) with the adjuvant DYG. Tu et al. (15) also gained similar outcomes of more oocyte retrieval (*p* < 0.001) and top-quality embryos (*p* = 0.038) and a lower incidence of untimely LH rise (0.7% vs. 8.3%, *p* = 0.001) in DOR patients.

#### 3.1.6 Comparison with the natural cycle

Chen et al. (16) investigated the inhibitory capacity of the natural cycle and PPOS protocol on the LH peak. Employing mild stimulation (MPA 10 mg/d from MC 3, hMG 75–150 U when FSH was ≤ 8 U/L) in this study, the MPA group achieved statistically lower incidence of spontaneous ovulation (2% vs. 10.8%) and premature LH surge (1% vs. 50%), and significantly more recovered oocytes and viable embryos. The authors certificated the robust inhibition of MPA on LH surge, with no side effects on oocyte quality and embryo competence.

### 3.2 Incidence of premature LH surge and OHSS risk

Several studies showed stronger control of the PPOS scheme over premature LH surge regardless of the progestin type. Kuang et al. (10) reduced the spontaneous LH surge rate to 0.7% with MPA, which is significantly lower than the reported 0.34% – 38% in the GnRHant group. Zhu et al. (18) compared Duphaston (20 mg/d) vs. Utrogestan (100 mg/d), while Yu et al. (31) compared Duphaston (20 mg/d) vs. MPA (10 mg/d) respectively, in similarly designed prospective studies in normal ovulatory patients. Similar results were obtained: no premature LH surge, no moderate or severe ovarian hyperstimulation syndrome (OHSS), and similar laboratory data and pregnancy rates in each study. In a Japanese study (20), no patient had a spontaneous LH peak, while one patient developed moderate OHSS in PPOS and GnRHant groups, respectively. As the primary endpoint of a prospective RCT in POR patients performed by Chen et al. (51), the premature LH surge was zero in the PPOS group vs. 5.88% in the antagonist group and neither group showed moderate or severe OHSS. Xiao et al. (52) established the reduced risk of mild-moderate OHSS (0 vs. 6.67%) in the MPA group, with no harmful effect on clinical results in PCOS patients compared with the GnRHant group. In a comparison of DYG and antagonist in another PCOS cohort (19), the results were consistent with Zhu et al. (18) and Yu et al. (31). A systematic review (53) also summarized PPOS protocol to result in a lower frequency of OHSS (RR 0.52, 95% CI 0.36–0.75) and equivalent pregnancy rates when compared with the controls.

### 3.3 Neonatal outcomes

After years of application, the safety of mild stimulation and GnRHa short protocol have been verified (48, 54, 55). Zhang et al. (56) enrolled 4,596 newborns in a retrospective study to investigate the potential impact of PPOS and the two conventional protocols on fetal development and calculated no difference in neonatal outcomes or congenital malformations among the three arms. Wang et al. (57) investigated 1,589 infants from either the PPOS regimen or GnRHa short protocol and confirmed similar birth characteristics and live-birth defects rate. Liang et al. (58) investigated infants delivered by mothers with advanced endometriosis who underwent ART with either PPOS, GnRHant protocol, or GnRHa long protocol and failed to exhibit any difference in neonatal outcomes. No adverse effect of progestin on embryonic development potential was established.

### 3.4 Cost-effectiveness

Longer duration and higher dosage of Gn are common profiles in PPOS protocol than others except for GnRHa downregulation protocol due to its stable control of the pituitary (10, 15, 20, 33, 50, 53). The PPOS protocol is still more economical during COH because of the lower price of progestin than GnRH analog. A study (20) calculated a total cost savings of 176.5 dollars in inhibiting premature LH peak with DYG over antagonist per patient. Mathieu et al. (59) stated that the PPOS scheme could save 209 euros per aspirated cycle. However, the cryopreservation of embryos and freeze-thaw procedure necessitated for PPOS will increase the overall cost, which makes it less cost-effective in that sense (60). Overall, this protocol may be especially applicable for those who attempt fertility preservation, preimplantation genetic diagnosis, or oocyte donors, or those with endometrial polyps and need treatment before transfer (61).

## 4 Discussion

Premature LH surge occurs in 3% – 10% of IVF cycles, it compromises oocyte yield and leads to embryo-endometrium asynchrony and hence increases cycle cancelation rate (2, 3, 38, 62). Conventional protocols employ GnRHa or antagonist to prohibit the premature LH rise, however, the GnRHa protocol is prone to high OHSS risk, hypo-oestrogenic symptoms, and ovarian cysts (4, 63), while the antagonist protocol is associated with decreased oocytes yield, increased cancellation rate, and incomplete LH surge blockade (5, 6, 41, 62), especially for those with advanced age or DOR (7, 8). Besides, both increase the complexity and cost of treatment. So, specialists come up with the novel use of progestin.

Progestin has been used for contraception for at least 50 years, it blocks the E2-induced LH surge to inhibit ovulation (64). Based on this, experts explored its application in preventing preovulation (10, 31, 35). High concentration of progestin can suppress the hypothalamic GnRH pulse, thus reducing the pulsatile secretion of LH (65). Follicular phase administration of progestin generates a decrease in the frequency and amplitude of LH pulses, as well as a reduction in plasma concentration (66). It is assumed that three stages constitute the GnRH surge: initiation, transmission, and release in a natural cycle (65). The adjuvant progestin in the stages of signal initiation and transmission can prohibit the surge, however, it will not work if given during the late stage of signal transmission, i.e., the onset of GnRH/LH surge (67). So the role of progestin in stimulating or blocking an LH surge depends on its administration time (18). Therefore, it is scheduled during the early follicular phase in PPOS protocol, or when the E2 level is less than 50 – 70 pg/mL (10).

GnRH-ant causes rapid suppression of LH by competing for GnRH receptors (68), but endogenous estrogen can induce some GnRH release, so a premature LH surge may still occur (51). In the PPOS protocol, the application of progesterone precedes the estrogen surge, inhibiting hypothalamic GnRH pulses through negative feedback and thereby indirectly and moderately controlling the pituitary, maintaining a stable low level of LH throughout the COH process and ensuring oocyte maturation and quality (51, 69, 70). However, accurately comparing different studies on premature LH surge is challenging. Firstly, there is a lack of a precise definition: some consider an increase in LH levels over 10 (59, 71, 72) or 15 (51, 73) as premature, while others only intervene when LH levels exceed 20 (16). Secondly, there are differences between protocols, for example, even within antagonist protocols, the starting time, dosage, and frequency of antagonists vary.

In terms of laboratory results and pregnancy outcomes, most studies suggest that PPOS is comparable to conventional regimens, including rates of aneuploidy, number of oocytes retrieved, number of top-quality embryos, CPR, LBR, and miscarriage rate (12, 74–76). Previous studies have suggested that LH accumulation during the follicular phase may impair GCs, reduce the developmental potential and maturity of oocytes, leading to suboptimal oocytes and embryos (77–79). The PPOS protocol can antagonize LH surges by reducing the frequency of LH pulses and increasing the concentration of E2 and P in follicular fluid, which provide a suitable internal environment for oocyte maturation (80, 81). Carter et al. (82) observed not only no effect of exogenous progestin on *in vitro* oocyte maturation and blastogenesis but progestin also enhanced fertilization and cleavage rate. Aparicio et al. (83) reported a positive correlation between exogenous P and oocyte quality.

Of course, experts currently have concerns about this new scheme. Some believe that progesterone may cause the developmental arrest of oocytes in the GV stage, reducing the blastocyst rate. Salehnia and Zavareh (84) suggested that progestin will reduce oocyte maturity rate if the concentration of adjuvant P increased from 10 to 100 µmol/L. Silva and Knight (85) supported this view and reported a 40% decrease in bovine blastocyst rate with the addition of progestin. Progesterone receptors are expressed in granulosa cells, and progesterone exerts its effects on oocytes through them (86). Some scholars (87) have pointed out that high levels of P before ovulation disrupt the mitochondrial OXPHOS pathway, damaging granulosa cell function and consequently harming oocyte quality. The administration of antioxidants can reverse this effect. Therefore, for patients at risk of oxidative stress, such as those who are obese or elderly, PPOS may not be the best option (86). Thus the impact of the PPOS scheme on oocyte and embryo development requires more in-depth exploration through both *in vivo* and *in vitro* studies.

OHSS is one of the common complications in IVF, usually mild to moderate (88). Due to the development of multiple follicles, several corpora lutea formed when exogenous hCG was used to induce the final maturation of oocytes, which stimulates the ovaries to produce a large amount of vascular endothelial growth factor (VEGF) and other vasoactive substances, increasing vascular permeability, which causes fluid accumulation outside the blood vessels (89, 90). The endogenous and exogenous hCG after embryo transfer exacerbate this pathology, and in severe cases, it can be life-threatening. Unlike the pituitary-suppressing effect of the long GnRHa protocol, the PPOS regimen is allowed to use GnRHa to trigger the final maturation of oocytes. The advantage of GnRHa is that it simultaneously triggers LH and FSH surges, mimicking the endocrine characteristics of the natural cycle, thereby promoting oocyte maturation and cumulus expansion (91). In addition, the short-term LH/FSH surge by GnRHa trigger reduces luteal VEGF secretion, preventing OHSS (92). It has been reported that in IVF cycles, the OHSS rate triggered by HCG is around 16% – 30%, whereas GnRHa significantly reduces this proportion (93). Therefore, PPOS combined with GnRHa trigger is an ideal choice for reducing the risk of OHSS and maintaining pregnancy potential. At the same time, the inherent freeze-all strategy of the PPOS protocol separates the sequential processes of ovarian stimulation and embryo transfer, which effectively reduces the incidence of OHSS (90), making it a suitable choice for populations at high risk of OHSS, such as those with PCOS.

Compared with conventional GnRH analog regimen (11, 21, 46, 94, 95), natural cycle (16, 61), or mild stimulation cycle (16, 27, 57, 63), whether they were in poor (13, 16, 30, 44, 45, 96), normal (10, 18, 66, 97), or high responders (17, 52, 94, 97), RCT or not (53, 98), prospective and retrospective (10, 21, 53, 96, 99), or whether the primary measurement were ovarian stimulation characteristics (85, 98, 99), endocrinological profiles (46, 51, 65–67), embryological features (16, 27, 84), clinical results (28, 43, 52, 57), neonatal outcomes (56–58), or incidence of preovulatory LH surge and OHSS (12, 19, 35, 51, 65), PPOS has achieved encouraging results. In summary, there are mainly two viewpoints: one advocates that the PPOS scheme shows no significant statistical difference from the control group, and is even superior to the control group in aspects such as the suppression of the early LH peak and the prevention of OHSS. The other viewpoint suggests that the advantages of PPOS are not universal, as patients who are older or have endometriosis may not benefit significantly.

This article suffers from some limitations. Firstly, the efficacy of different types and doses of progestin, as well as modifications of the PPOS protocol, were not discussed. Secondly, differences in type and initial dosage of Gn, ovulation trigger agent, the threshold of premature LH surge, etc., may affect the evaluation power and needs to be refined. Well-designed and more comprehensive future studies are warranted to investigate the strengths and weaknesses of the PPOS protocol.

## 5 Conclusion

In conclusion, the PPOS protocol is not inferior when compared with the controls and is an effective and safe choice for ART. In the future, the benefits and drawbacks of this program, as well as the target population, need to be supported by more high-quality clinical research.
