# Supplementary material for: Progestin-primed ovarian stimulation protocol in patients undergoing assisted reproductive technology
Source: Front Reprod Health. 2026 Jan 23;7:1719930. doi: 10.3389/frph.2025.1719930 (PMC12876159; doi:10.3389/frph.2025.1719930)
Supplement: Supplementary file 1 [file Table1.docx]

| Author | Year of publication | Research classification | Characteristics of the research population | In comparison with | Involved progestin type | Main outcome | The difference results compared to the control group |
| --- | --- | --- | --- | --- | --- | --- | --- |
| Huang et al. | 2018 | Retrospective study | POR | GnRH-ant protocol | MPA 10 mg/d | Clinical pregnancy rates, abortion rates, serum LH and E2 levels on hCG day, MII oocyte rates, fertilization rates, good-quality embryos | MII oocyte rate↑, fertilization rate↑, Good-quality embryo rate↑, CPR↑, LBR↑ |
| Eftekhar et al. | 2019 | RCT | PCOS | GnRH-ant protocol | DYG 20 mg/d | Pregnancy outcomes: IR, BR, CPR, miscarriage rate | No. of MII oocytes↓，Maturity rate↓，Maturity rate↓，Total cycle cancelation↑，Total cycle cancelation↓，LH levels of on trigger↑，OHSS (mild and moderate)↓ |
| Xi et al. | 2020 | RCT | NOR | GnRHa-long protocol | MPA 4mg/d | Retrieved oocytes | Gn duration and dose↓ |
| Liang et al. | 2020 | Retrospective study | Patients with advanced endometriosis | GnRH-ant protocoland GnRHa protocol | MPA 10 mg/d | Neonatal outcomes; Congenital malformations | The indicators involved had no statistical differences. |
| Giles et al. | 2021 | RCT+retrospective study | Oocyte donors and recipients | GnRH-ant protocol | MPA 10 mg/d | Primary outcome: No. of oocytes and MII oocytes retrieved; Secondary outcomes: incidence of early ovulation and OHSS, Gn dose and time, E2, P4, and LH of serum and follicular fluid, fertilization rate, CPR, OPR, and LBR | The indicators involved had no statistical differences. |
| Caetano et al. | 2021 | Retrospective study | NOR | GnRH-ant protocol | MPA 10 mg/d | Primary outcome: CPR; Secondary outcome: mean MII oocytes retrieved,  fertilization rate, blastocyst formation rate, mean duration  of stimulation and dose of Gn | MII oocytes↓ |
| Zhao et al. | 2022 | Retrospective study | NOR | GnRH-ant protocol and LPS | MIP 300 mg/d | Primary outcome: No. of retrieved oocytes; Secondary outcome: No. of vitrified oocytes or embryos, Gn duration and total dose,  peak E2 levels, LH surge rate, COS cost | Total dose of Gn and duration of stimulation↓ vs. LPS group; E2 at trigger day↑ vs. the other two groups; injection and COS cost↓ vs. GnRH-ant group |
| Kao et al. | 2022 | Retrospective study | POR | GnRH-ant protocol | MPA 10 mg/d | primary outcome: incidence of premature LH surge; Secondary outcomes: No. of oocytes retrieved, rates of oocyte retrieval, and rates of MII oocytes | Duration of progestin↑, premature LH surge↓ |
| Shibasaki et al. | 2023 | Retrospective study | NOR | GnRH-ant protocol | CMA | Embryo developments, clinical results, or neonatal outcomes | Total Gn dose↑, triggered LH level↓, triggered P level↓ |
| Cai et al. | 2024 | RCT | Predicted suboptimal response patients | GnRH-ant protocol | MPA 10 mg/d | Primary outcome: LBR; Secondary outcomes: incidence of premature LH surge and cycle cancellation, No. of retrieved oocytes or fertilized oocytes, No. of viable and high-quality embryos, No. of cryopreserved embryos, time to live birth | LH level on trigger day↑, endometrial thickness on trigger day↓ |
| Vidal et al. | 2024 | Prospective study | NOR | GnRH-ant protocol | MIP 200mg/d | primary outcome: No. of euploid embryos; Secondary outcomes: the endocrine profile, No. of oocytes and MIIs retrieved, fertilization rate, blastocyst formation rate | E2, P and LH on triggering day↑, No. of COCs, MII and 2PN↑ |
| Kuang et al. | 2015 | Prospective study | NOR | GnRH-a short protocol | MPA 10 mg/d | Primary outcome: No. of oocytes retrieved; Secondary outcomes: No. of mature oocytes, incidence of premature LH surge, clinical pregnancy outcomes | Gn dose and duration↑, oocyte retrieval rate↑ |
| Zhu et al. | 2015 | Retrospective study | NOR | GnRH-a short protocol | MIP 200mg/d | Primary outcome: No. of oocytes retrieved; Secondary outcomes: No. of mature oocytes, incidence of premature LH surge, clinical pregnancy outcomes | Gn dose and duration↑，No. of >10 mm follicles↑, No. of >14 mm follicles↑, No. of viable embryos and Viable embryo rate↑ |
| Wang et al. | 2016 | RCT | PCOS | GnRH-a short protocol | MPA 10 mg/d | OPR | Gn dose↑, OPR↑ |
| Chen et al. | 2017 | Prospective study | DOR | Natural cycle | MPA 10 mg/d | Primary outcomes: incidence of spontaneous LH surge and premature ovulation; Secondary outcomes: steroid hormone profiles, No. of retrieved oocytes and viable embryos | No. of >10 mm follicles↑, No. of >14 mm follicles↑, No of retrieved oocytes and fertilized oocytes↑ |
| Zhang et al. | 2017 | Retrospective study | Children | GnRH-a short protocol and mild stimulation | MPA 10 mg/d | Neonatal outcomes and congenital malformations | The indicators involved had no statistical differences. |
| Iwami et al. | 2018 | Retrospective study | NOR | GnRH-ant protocol | DYG 20mg/d | Primary outcome: OPR; Secondary outcomes: No. of COCs, No. of viable embryos, fertilization rate, CPR, early miscarriage rate | Gn dose and duration↑, LH on the trigger day↓ |
| Wang et al. | 2018 | Retrospective study | Infants | GnRH-a short protocol | MIP 100mg/d | Neonatal outcomes and risk of congenital malformations | Multiple delivery cycles↑ |
| Chen et al. | 2019 | RCT | POR | GnRH-ant protocol | MPA 10 mg/d | Primary outcome: incidence of premature LH surge; Secondary outcomes: No. of oocytes and viable embryos, CPR,IR, LBR | LH values on trigger day↓, twin pregnancy rate↑ |
| Peng et al. | 2019 | Retrospective study | Women with ages ≥40 years | Mild stimulation protocol | DYG 20mg/d | Primary outcome: top quality embryo rate; Secondary outcome: CPR | Gn dose↑, LH, E2, and P on trigger day↓, premature LH surge↓, top-quality embryos rate↑ |
| Beguería et al. | 2019 | RCT | Oocyte donors and recipients | GnRH-ant protocol | MPA 10 mg/d | Primary outcome: No. of MII oocytes retrieved; Secondary outcomes: embryological laboratory outcomes and reproductive outcomes | BR↓, CPR↓, OPR↓ |
| Xiao et al. | 2019 | Prospective study | PCOS | GnRH-ant protocol | MPA 10 mg/d | Ovarian response and clinical outcomes | Gn dose and duration↑, E2 level at HCG day↓, retrieved oocytes↓, cryopreserved embryos↓, mild-to-moderate OHSS↓ |
| Argent et al. | 2020 | Prospective study | Patients with endometriosis | GnRH-ant protocol | promegestone , chlormadinone, medrogestone , acetate of nomegestrol, or desogestrel | Primary outcome: No. of oocytes retrieved; Secondary outcomes: No. of vitrified oocytes, rate of moderate/severe OHSS, rate of cycle cancellation, LH surge incidence | LH level↓ |
| Du et al. | 2021 | Retrospective study | POR | GnRH-ant protocol | MPA 6 mg/d | Primary outcome: CLBR; Secondary outcome: No. of retrieved oocytes , No. of 2PN embryos and available embryos | Dosage of Gn↑, time to live birth↑ |
| Tu et al. | 2021 | Retrospective study | DOR | Mild stimulation protocol | MPA 10 mg/d or DYG 20mg/d | Primary outcomes: CCPR and CLBR; Secondary outcomes: No. of oocytes retrieved and top-quality embryos | Total doses and duration of Gn↑, LH, P on trigger day↓, E2 on trigger day↑, premature LH surge rate↓, No. of oocytes retrieved↑, No. of normal fertilization↑, No. of top-quality embryos↑ |
| Huang et al. | 2021 | Retrospective study | PCOS | GnRH-ant protocol | DYG 20mg/d | Primary outcome: the duration of reduction in GnRHant injections; Secondary outcomes: incidence of premature LH surge and OHSS, additional duration/dose of Gn, No. of retrieved oocytes and frozen embryos, fertilization rate, IR, CPR, LBR | Duration of GnRHant injections↓ |
| Yang et al. | 2022 | Retrospective study | Patients with endometrioma | Ultra-long GnRHa protocol and GnRH-ant protocol | MPA 10 mg/d | Primary outcome: LBR; Secondary outcomes: IR, BR, CPR, OPR | Gn dose and duration↓ vs. ultra-long, No. of retrieved oocytes ↓ vs. ultra-long, No. of viable embryos↓ vs. ultra-long and GnRH-ant protocol, thickness of endometrium↓ vs. ultra-long，IR，BR，CPR，LBR↓ vs. ultra-long, ongoing miscarriage↓ vs. ultra-long, late miscarriage vs. ultra-long |
| Kalafat et al. | 2022 | Retrospective study | NOR | GnRH-ant protocol | MPA 10 mg/d | No. of MII oocytes, rates of empty follicle syndrome, maturation, fertilization, blastulation, and cumulative clinical pregnancy | Duration of stimulation↑, E2 levels at maturation trigger↑, follicle >14 mm at maturation trigger↑ |
| Turkgeldi et al. | 2020 | Retrospective study | DOR | GnRH-ant protocol | MPA 10 mg/d | Primary outcomes: No. of MII oocytes retrieved and premature LH surge events; Secondary outcomes: duration and total consumption of Gn, No. of oocytes retrieved, and oocyte maturation rate | The indicators involved had no statistical differences. |
