# Supplementary figures and images for: Progestin-primed ovarian stimulation protocol in patients undergoing assisted reproductive technology
Source: Front Reprod Health. 2026 Jan 23;7:1719930. doi: 10.3389/frph.2025.1719930 (PMC12876159; doi:10.3389/frph.2025.1719930)

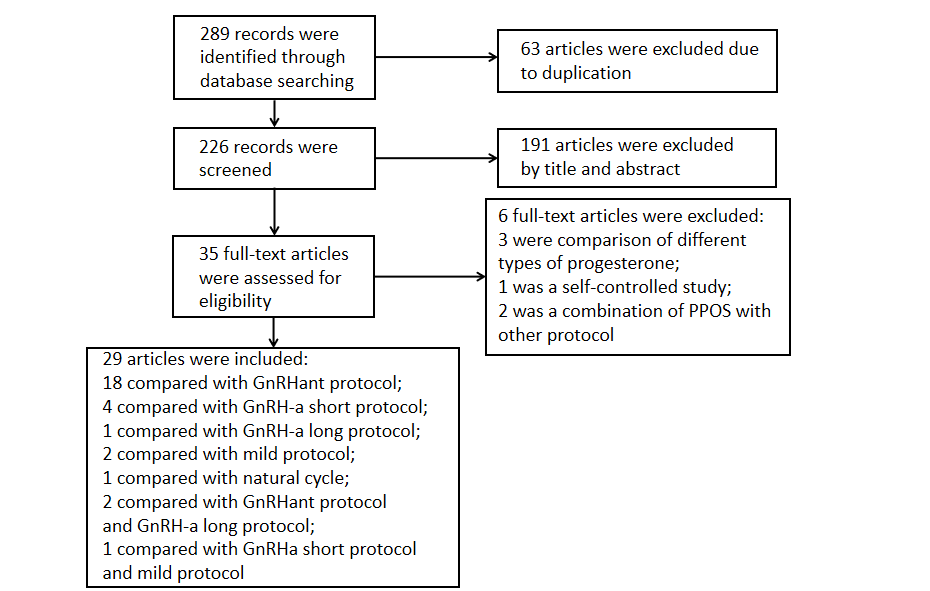

Supplement: Supplementary file 2 [file Image1.png]
